# Safety and Pharmacokinetics of Intravenous and Oral Fosmanogepix, a First-in-Class Antifungal Agent, in Healthy Volunteers

**DOI:** 10.1128/aac.01623-22

**Published:** 2023-03-29

**Authors:** Michael R. Hodges, Eric Ople, Pamela Wedel, Karen J. Shaw, Abhijeet Jakate, William G. Kramer, Sjoerd van Marle, Ewoud-Jan van Hoogdalem, Margaret Tawadrous

**Affiliations:** a Amplyx Pharmaceuticals, Inc., San Diego, California, USA; b Hearts Consulting Group, LLC, Poway, California, USA; c Pfizer Inc., Groton, Connecticut, USA; d Kramer Consulting, LLC, North Potomac, Maryland, USA; e ICON, Groningen, the Netherlands

**Keywords:** APX001, APX001A, clinical trials, fosmanogepix, Gwt1 inhibitor, intravenous, manogepix, multiple ascending doses, orally bioavailable, phase 1, single ascending dose

## Abstract

Fosmanogepix (FMGX, APX001), a first-in-class, intravenous (i.v.) and oral (p.o.) antifungal prodrug candidate is currently in clinical development for the treatment of invasive fungal infections. Manogepix (MGX, APX001A), the active moiety of FMGX, interferes with cell wall synthesis by targeting fungal glycosylphosphatidylinositol-anchored cell wall transfer protein 1, thereby causing loss of cell viability. Data from two phase 1, placebo-controlled, single-ascending dose (SAD) and multiple-ascending dose (MAD) studies evaluating safety, tolerability, and pharmacokinetics of FMGX (doses up to 1,000 mg, i.v. and p.o.) are presented. Eligible participants were healthy adults (aged 18 to 55 years) randomized to receive either FMGX or placebo. Across both phase 1 studies, 151 of 154 participants (aged 23 to 35 years; FMGX: 116, placebo: 38) completed the study. Administration of FMGX i.v. demonstrated linear- and dose-proportional pharmacokinetics of MGX in terms of geometric mean maximum concentration of drug in serum (*C*_max_) (SAD: 0.16 to 12.0 μg/mL, dose: 10 to 1,000 mg; MAD: 0.67 to 15.4 μg/mL, dose: 50 to 600 mg) and area under the concentration-time curve (AUC) (SAD: 4.05 to 400, MAD: 6.39 to 245 μg · h/mL). With single and repeat p.o., dose-proportional increases in *C*_max_ (SAD: 1.30 to 6.41 μg/mL, dose: 100 to 500 mg; MAD: 6.18 to 21.3 μg/mL, dose: 500 to 1,000 mg) and AUC (SAD: 87.5 to 205, MAD: 50.8 to 326 μg · h/mL) were also observed, with high oral bioavailability (90.6% to 101.2%). Administration of FMGX p.o. under *post cibum* conditions improved tolerability versus *ante cibum* conditions. No severe treatment-emergent adverse events (TEAEs), serious AEs, or withdrawals due to a drug-related TEAEs were reported with single or multiple i.v. and p.o. doses. Preclinical target exposures were achieved and were not accompanied by any serious/unexpected concerns with generally safe and well-tolerated dose regimens.

## INTRODUCTION

The incidence of invasive fungal infections (IFIs) has increased globally due to a substantial rise in the number of immunocompromised patients, including organ transplant recipients, individuals with hematological malignancies or on prolonged immunosuppressive therapy, the elderly, and premature infants (1–4). In a study based on the 2013 National Health Interview Survey (NHIS), an estimated prevalence of self-reported immunosuppressed adults was 2.7% in the United States (5). Currently available antifungals are less than ideal due to limited spectrum of activity, emerging drug resistance, safety concerns, drug-drug interactions (DDIs), or poor tolerance (6–8). One of the difficulties in developing safe and well-tolerated antifungal agents is the similarity between fungi and human proteins and functions. Thus, drug targets must be chosen that are fungal specific (e.g., cell wall), or new molecules must demonstrate fungal specificity against a shared eukaryotic target (2, 9). Thus, there is an urgent need to overcome challenges associated with currently approved therapies by developing new antifungals.

Fosmanogepix (FMGX, APX001/PF-07842805; formerly E1211) is the first member of the “gepix” class of antifungals and is a small-molecule intravenous (i.v.) and oral (p.o.) water-soluble phosphate prodrug under clinical development for the treatment of IFIs. FMGX is quickly and extensively metabolized by systemic alkaline phosphatases to the active moiety, manogepix (MGX, APX001A; formerly E1210) (10). The mechanism of action of MGX is novel. It inhibits glycosylphosphatidylinositol (GPI)-anchored wall transfer protein 1 (Gwt1), a highly conserved fungal enzyme essential for cell viability. Inhibition of Gwt1 impairs the maturation and localization of cell wall mannoproteins required for cell wall integrity, adhesion, pathogenicity, and evasion of host recognition (11, 12). MGX is highly selective and has no inhibitory activity against the closest human ortholog of Gwt1, phosphatidylinositol glycan anchor biosynthesis class W (PIG-W) protein, consistent with the potential for a significant target-based safety therapeutic window (11).

MGX exhibits potent, broad-spectrum antifungal activity *in vitro* against clinically important strains of *Candida*, Cryptococcus, Aspergillus, and other rare molds, including *Scedosporium*, Lomentospora prolificans, and Fusarium spp. as well as members of the Mucorales order (8, 13–15). MGX maintains activity against target-based azole-, echinocandin- and amphotericin B-resistant organisms (10, 14).

The efficacy of FMGX has been examined in several *in vivo* models of IFIs, including disseminated (Candida albicans, Candida glabrata, Candida auris, Cryptococcus neoformans, Fusarium solani), and pulmonary (Aspergillus fumigatus, Aspergillus flavus, Scedosporium prolificans, Rhizopus arrhizus var. *delemar* and R. arrhizus var. arrhizus) models (8, 10, 16–19). In addition to demonstrating increased survival, several murine and rabbit infection models demonstrated reduced fungal burden in lung, kidney, eye, spinal cord, and brain (15, 20). MGX penetrates the central nervous system, unlike the echinocandins, which are recommended as first-line therapy for the treatment of invasive candidiasis, including candidemia, by the Infectious Diseases Society of America (IDSA) (15, 21–24).

Development of FMGX has progressed to evaluations in a clinical phase 3 setting. Here, we present the pharmacokinetics (PK), safety, and tolerability of i.v. and p.o. FMGX formulations in healthy adult volunteers from the first two completed phase 1 clinical studies of FMGX.

(The results of these studies were presented in part at IDWeek 2017, San Diego, CA [25, 26].)

## RESULTS

### Participant demographics and baseline characteristics.

A total of 154 healthy participants were randomized to treatment (FMGX: 116, placebo: 38) across both phase 1 studies. This excludes 12 participants from the open-label cohort (cohort 4) since it focused on a different aspect (assessment of DDI potential of FMGX) and will be reported separately. A total of 151/154 participants completed treatments; 2 withdrew consent and 1 withdrew due to moderate pyrexia (considered unrelated to the study drug). Gender distribution varied across cohort groupings and ranged from 30% to 100% male. Participants in each cohort grouping were mostly white (75% to 100%), young adults (23 to 31 years) of normal weight as determined by body mass index (BMI: 22.1 to 24.3 kg/m^2^) (Table 1).

**TABLE 1:** Participant demographics and baseline characteristics (safety population)^*a*^

	Study 1 cohorts (i.v.)				Study 2 cohorts (i.v. and p.o.)		
Parameter	1–6 (*n* = 48)	7–10 (*n* = 32)	11a–11d (*n* = 32)	12 (*n* = 8)	1a (*n* = 8)	1b (*n* = 10)	2–3 (*n* = 16)
Sex (% male)	63	59	47	63	100	30	38
Race (% white/black/Asian/other)	85/2/6/6	78/9/6/6	75/6/0/19	100/0/0/0	88/0/13/0	90/0/0/10	88/13/0/0
Age, mean ± SD (yrs)	27 ± 10	30 ± 9	27 ± 9	23 ± 4	35 ± 14	27 ± 8	31 ± 13
BMI, mean ± SD (kg/m^2^)	23.1 ± 2.4	23.5 ± 2.3	23.2 ± 3.0	22.1 ± 1.6	24.3 ± 2.0	22.3 ± 2.0	23.6 ± 2.8

a BMI, body mass index; i.v., intravenous; *n*, number of participants; p.o., oral dose.

### Pharmacokinetics.

**FIG 1:**
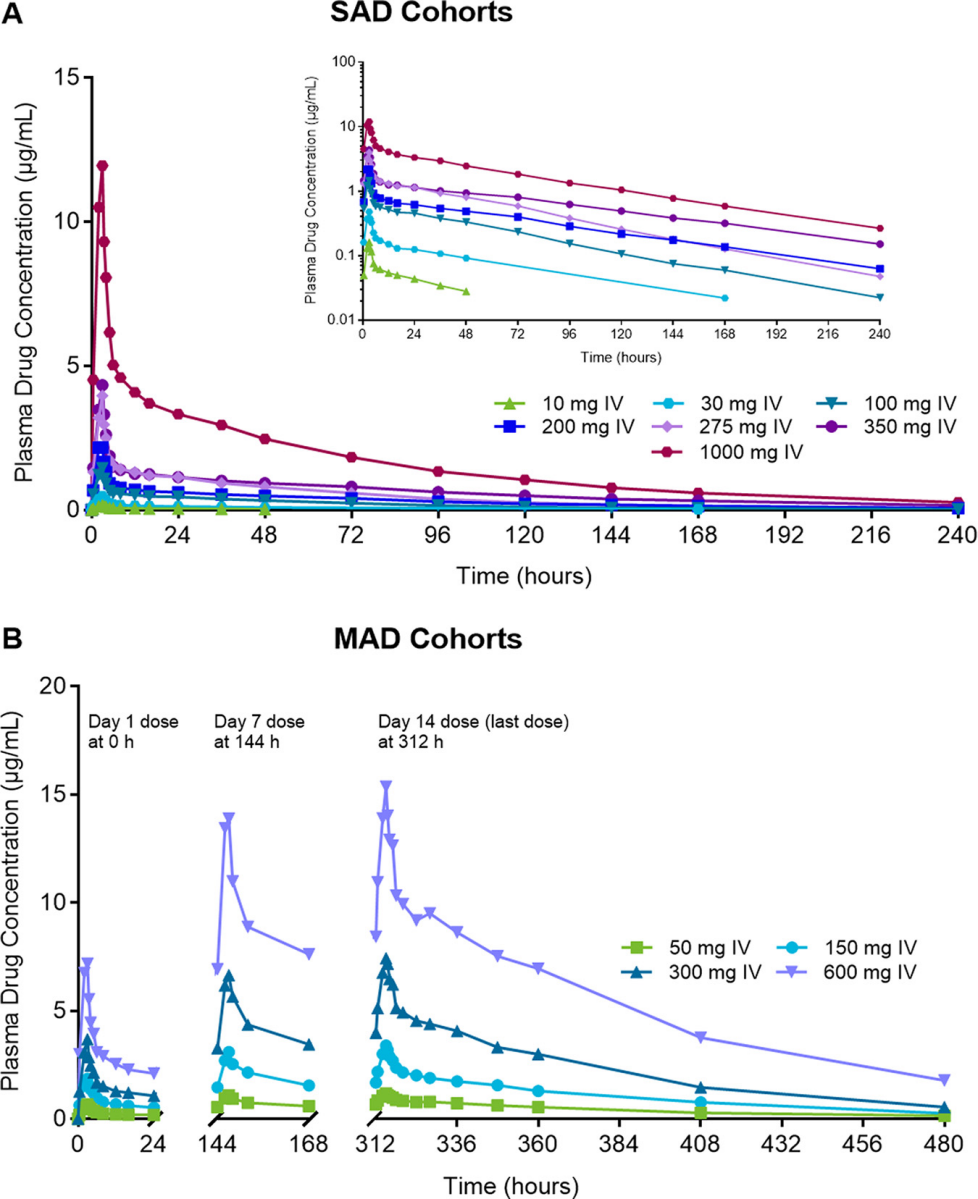
MGX concentration profile after single and multiple ascending i.v.-infused FMGX doses. (A) SAD cohorts; (B) MAD cohorts. (A and B) Data are presented for study 1 cohorts 1 to 6 and 11a (A) and cohorts 7 to 10 (B) as geometric mean MGX plasma concentrations for participants in the PK population (*n* = 5 for 50 mg, *n* = 6 for all other treatment groups) using linear (panel A, main figure and panel B) and semilogarithmic (panel A, inset) scales. All doses were infused over 3 h; doses were administered daily for the MAD segment. For panel A, the elimination half-life for the 10-mg dose was excluded from overall data interpretation because it was biased by the sampling schedule at that first dose level. FMGX, fosmanogepix; IV, intravenous; MAD, multiple ascending dose; MGX, manogepix; n, number of participants; PK, pharmacokinetics; SAD, single ascending dose.

**FIG 2:**
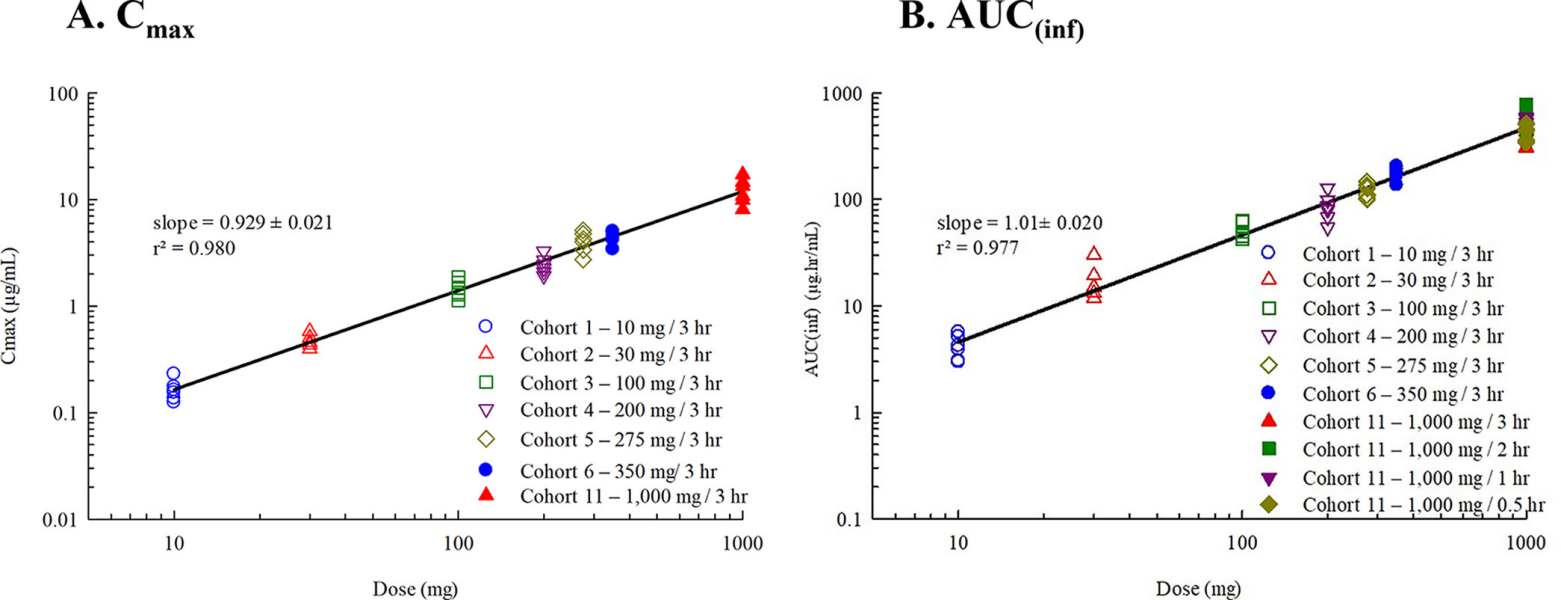
(A and B) Relationship of (A) *C*_max_ and (B) AUC_(∞)_ of MGX with dose after i.v. infusion of FMGX (SAD cohorts). Data are presented for study 1 cohorts 1 to 6 and 11a for *C*_max_ (A) and AUC_(∞)_ (B) of MGX for participants in the PK population. All doses were infused over 3 h (A) or 10 to 350 mg for 3 h and 1,000 mg over 0.5 to 3 h. AUC(_∞)_, area under the concentration-time curve from time zero to infinity; *C*_max_, maximum plasma concentration; FMGX, fosmanogepix; IV, intravenous; MGX, manogepix; n, number of participants; PK, pharmacokinetics.

**FIG 3:**
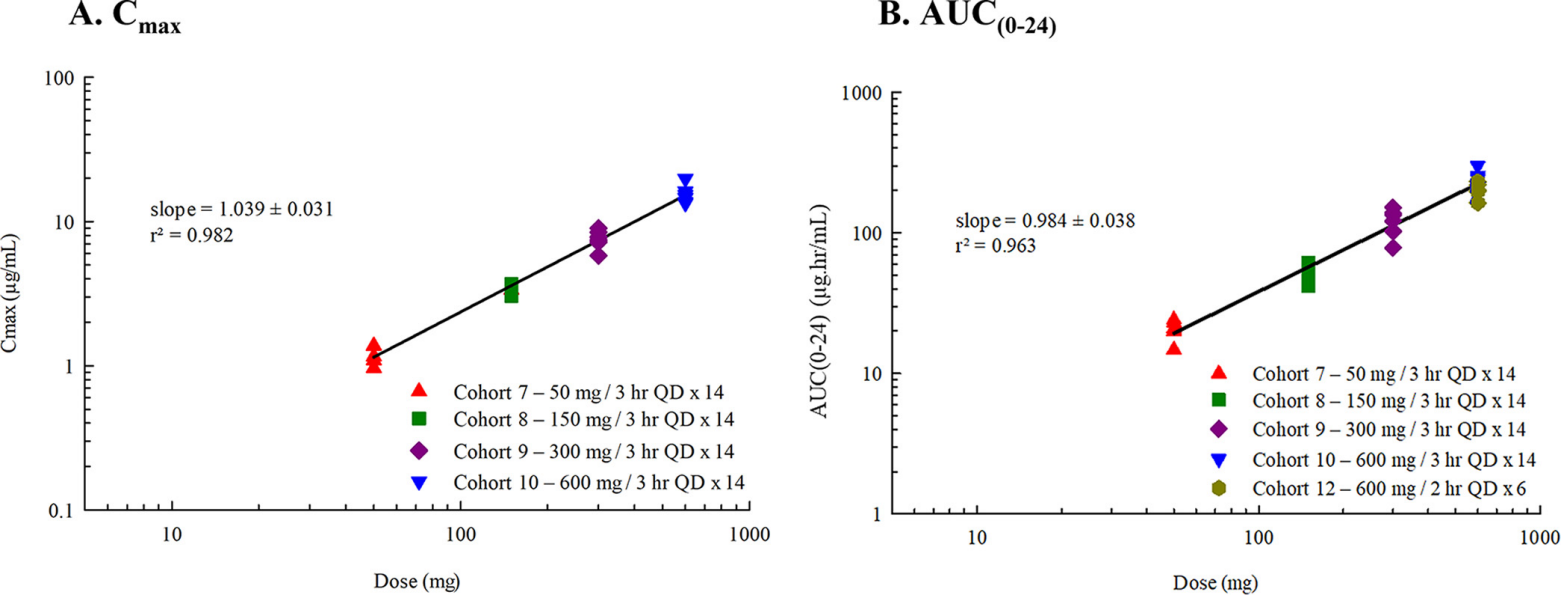
(A and B) Relationship of (A) *C*_max_ and (B) AUC_(0–24)_ of MGX with dose after i.v.-infused FMGX (MAD cohort). Data are presented for study 1 cohorts 7 and 8 for *C*_max_ (A) and AUC_(0–24)_ (B) of MGX for participants in the PK population. All doses were infused over 3 h QD × 14 days (A) or over 3 h QD × 14 days and on day 7 after i.v. infusion of 600 mg QD × 6 days preceded by 1,000 mg/1 h × 2 on day 1 (B). AUC_0–24_, area under the concentration-time curve from time 0 to 24 h postdose; *C*_max_, maximum plasma concentration; FMGX, fosmanogepix; IV, intravenous; MAD, multiple ascending dose; MGX, manogepix; n, number of participants; PK, pharmacokinetics; PO, oral; QD, once daily.

**(i) Pharmacokinetics of single and multiple ascending i.v. FMGX doses.** A dose-proportional increase in the geometric mean plasma concentration of the active moiety MGX was consistently observed after single and multiple infusions of the prodrug FMGX over 3 h across all doses tested, ranging from single doses of 10 mg to 1,000 mg and multiple doses of 50 mg to 600 mg administered once a day (QD) over 14 days (Fig. 1). Consistent with the PK profiles, the geometric mean values for maximum concentration of drug in serum (*C*_max_) and area under the concentration-time curve (AUC) also increased in a linear manner with both single and multiple ascending doses (Table 2). The *C*_max_ and AUC for single-ascending dose (SAD) cohorts 1 to 6 and 11 (Fig. 2), and multiple-ascending dose (MAD) cohorts 7 to 10 (Fig. 3) are illustrated.

**TABLE 2:** MGX pharmacokinetic parameters after single and multiple ascending i.v. infused FMGX doses^*a*^

Study 1 (i.v.) SAD cohorts								
Cohort			Study day	Pharmacokinetic parameter (geometric %CV)				
No.	FMGX dose (mg i.v.)	*n*		*C*_max_ (μg/mL)	AUC_∞_ (μg · h/mL)	*t*_1/2_ (h)	CL (mL/h/kg)	*V*_z_ (L/kg)
1	10^*b*^	6	1	0.16 (21.6)	4.05 (26.9)	39.2 (32.9)	27.0 (31.8)	1.53 (29.4)
2	30	6	1	0.48 (13.5)	16.7 (34.7)	61.0 (39.7)	19.6 (24.8)	1.72 (21.4)
3	100	6	1	1.44 (18.5)	50.4 (17.9)	52.5 (41.4)	24.8 (17.3)	1.88 (28.9)
4	200	6	1	2.41 (19.3)	83.2 (30.1)	67.0 (24.3)	25.5 (25.2)	2.46 (14.2)
5	275	6	1	3.96 (23.7)	120 (15.6)	48.6 (33.2)	25.0 (12.0)	1.75 (32.2)
6	350	6	1	4.33 (14.0)	173 (14.1)	74.9 (29.3)	20.9 (14.6)	2.26 (24.9)
Study 1 (i.v.) MAD cohorts								
Cohort			Study day	Pharmacokinetic parameter (geometric %CV)				
No.	FMGX dose (mg i.v.)	*n*		*C*_max_ (μg/mL)	AUC_0–24_ (μg · h/mL)	*t*_1/2_ (h)	CL (mL/h/kg)	*V*_z_ (L/kg)
7	50	5	1	0.67 (20.9)	6.39 (21.6)			
			7	1.09 (16.6)	17.6 (14.4)			
			14	1.18 (15.4)	20.3 (19.5)	69.0 (43.9)	24.5 (20.0)	2.44 (35.9)
8	150	6	1	1.87 (12.4)	19.4 (19.7)			
			7	3.09 (8.84)	48.7 (9.35)			
			14	3.42 (7.17)	51.9 (14.0)	52.6 (51.4)^*c*^	27.7 (17.3)	2.17 (36.9)^*c*^
9	300	6	1	3.69 (27.0)	37.1 (33.8)			
			7	6.65 (17.6)	104 (24.3)			
			14	7.52 (15.3)	118 (24.6)	53.1 (30.7)	29.0 (15.1)	2.22 (28.5)
10	600	6	1	7.57 (15.5)	72.7 (10.1)			
			7	14.5 (8.37)	217 (9.21)			
			14	15.4 (14.3)	245 (12.1)	64.8 (26.0)	29.4 (18.8)	2.75 (22.9)

a Data are presented for study 1 as the geometric mean (geometric %CV) for the PK population. All doses were infused over 3 h; doses were administered daily for the MAD segment. %CV, percent coefficient of variation; AUC_∞_, area under the concentration-time curve from time zero to infinity; AUC_0–24_, area under the concentration-time curve from time zero to 24 h postdose; *C*_max_, maximum plasma concentration; CL, clearance; FMGX, fosmanogepix; i.v., intravenous; *n*, number of participants; MAD, multiple ascending dose; MGX, manogepix; p.o., oral dose; SAD, single ascending dose; *t*_1/2_, terminal phase half-life; *V*_z_, volume of distribution.

b *n* = 5.

c The elimination half-life for the 10-mg dose was excluded from overall data interpretation because it was biased by the sampling schedule at that first dose level.

The median time to maximum concentration of drug in serum (*T*_max_) of 3.0 h for the single- and multiple dose regimens was concordant with the 3-h infusion time specific to these cohorts. Conversion to MGX was not the rate-limiting step since the geometric mean values for elimination half-life of the active moiety were consistent for all doses and regimens evaluated (48.6 to 74.9 h). Geometric mean values for systemic clearance (CL) were also consistent across doses and regimens (19.6 to 29.4 mL/h/kg).

As expected from the half-life (*t*_1/2_) and the dosing frequency, accumulation of MGX was observed with repeat dosing. Median accumulation values estimated as the ratio of either *C*_max_ or AUC from 0 to 24 h (AUC_0–24_) on day 14 to that on day 1 were 1.96 and 3.27, respectively, with no apparent trends among the multiple-dose regimens. Steady state was reached between day 7 and day 14, depending on the individual participant’s MGX *t*_1/2_ value.

### Pharmacokinetics of MGX over shortened i.v. infusion times.

**FIG 4:**
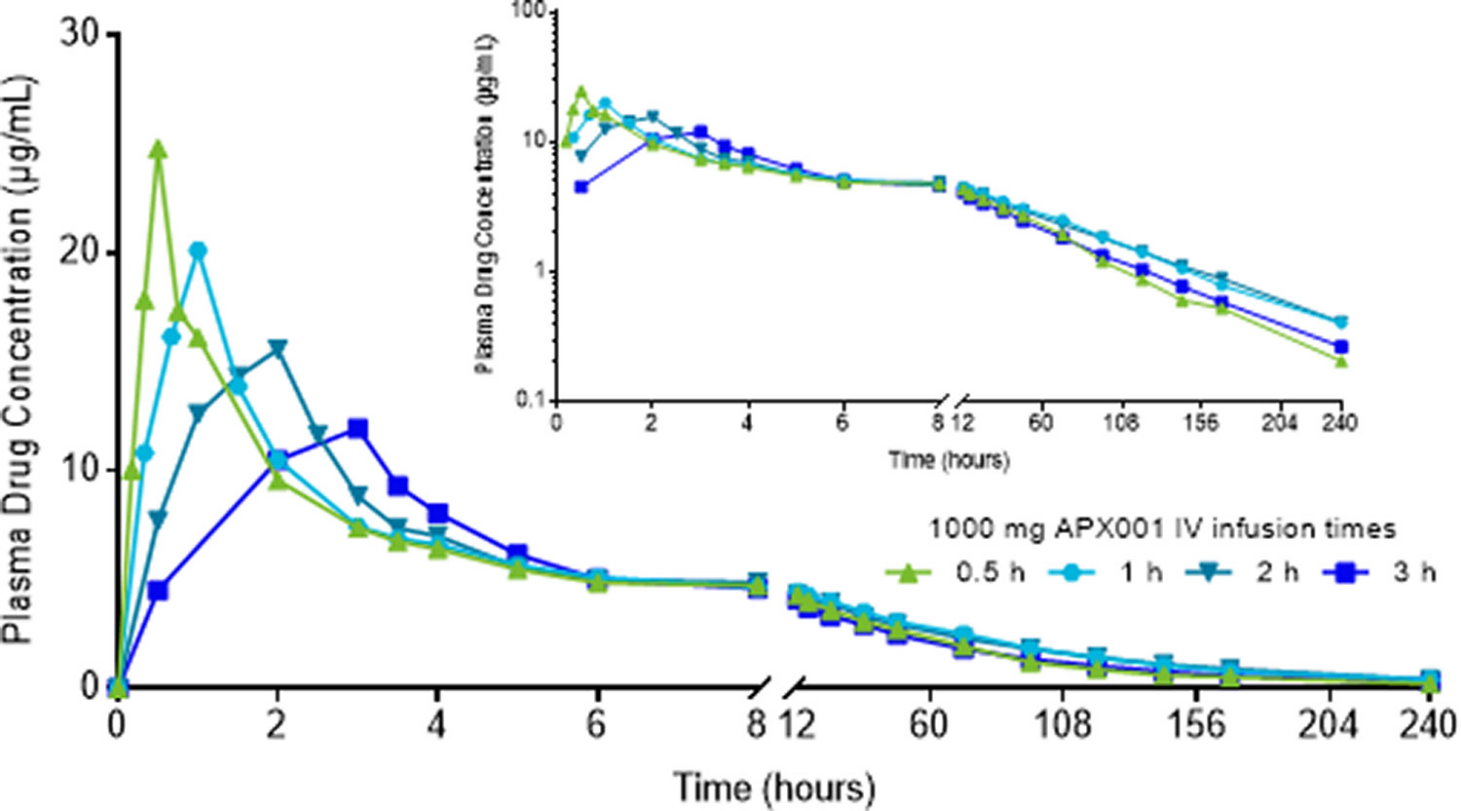
MGX single-dose concentration profile over shortened i.v. infusion times. Data are presented for study 1 cohorts 11a to 11d as geometric mean MGX plasma concentrations for participants in the PK population (*n* = 6 per group) using linear (main figure) and semilogarithmic (inset) scales. IV, intravenous; n, number of participants; MGX, manogepix.

After i.v. administration of single 1,000-mg FMGX doses over 0.5 to 3 h, peak MGX plasma concentrations increased and *C*_max_ occurred earlier as the infusion time was shortened (Fig. 4, Table 3), whereas AUC was consistent across the four groups (Table 3). The median *T*_max_ was concordant with the duration of infusion, and geometric mean values for CL, volume of distribution (*V*_z_), and *t*_1/2_ were within the ranges observed with other doses infused over 3 h as described above.

**TABLE 3:** MGX single-dose pharmacokinetic parameters over shortened i.v. infusion times^*a*^

Study 1 (i.v.) SAD cohort			Pharmacokinetic parameter (geometric %CV)				
No.	FMGX 1,000-mg i.v. infusion time (h)	*n*	*C*_max_ (μg/mL)	AUC_∞_ (μg · h/mL)	*t*_1/2_ (h)	CL (mL/h/kg)	*V*_z_ (L/kg)
11a	3	6	12.0 (28.6)	400 (20.9)	55.7 (38.3)	25.1 (16.7)	2.02 (39.9)
11b	2	6	15.7 (31.0)	541 (26.9)	72.5 (43.7)	23.2 (28.8)	2.43 (46.3)
11c	1	6	20.1 (17.5)	536 (9.24)	67.7 (37.6)	21.6 (15.6)	2.11 (28.4)
11d	0.5	6	24.9 (7.5)	434 (17.2)	55.6 (47.1)	24.6 (15.2)	1.97 (40.1)

a Data are presented for study 1 as the geometric mean (geometric %CV) for the PK population. %CV, percent coefficient of variation; AUC_∞_, area under the concentration-time curve from time zero to infinity; *C*_max_, maximum plasma concentration; CL, clearance; FMGX, fosmanogepix; i.v., intravenous; *n*, number of participants; MGX, manogepix; SAD, single ascending dose; t_1/2_, terminal phase half-life; *V*_z_, volume of distribution.

### Pharmacokinetics of MGX using a loading dose strategy.

**FIG 5:**
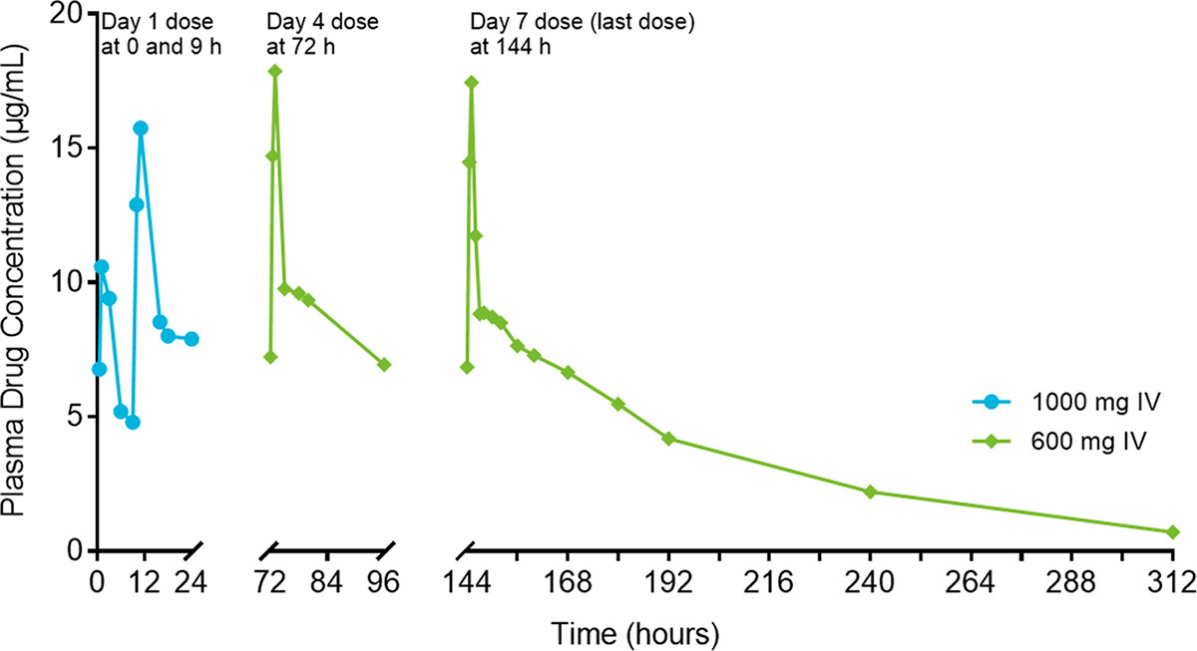
MGX concentration profile using a loading dose strategy. Data are presented for study 1 cohort 12 as geometric mean MGX plasma concentrations for participants in the PK population (*n* = 6) using linear a scale. A 1000-mg FMGX dose was infused over 2 h at 0 and 9 h on day 1 followed by 600-mg FMGX doses infused over 1 h once daily for 6 days (days 2 to 7). FMGX, fosmanogepix; IV = intravenous; n, number of participants; MGX, manogepix; PK, pharmacokinetics.

Treatment of IFIs is best achieved by reaching therapeutic exposures quickly. However, as illustrated in Fig. 1 and Table 2, steady state is reached between day 7 and day 14, consistent with the relatively long half-life of MGX. A loading regimen to safely reach steady-state exposures earlier was therefore investigated, based on simulations using data from previous cohorts, and evaluated in a separate cohort with a 7-day dosing period (Fig. 5, Table 4). The geometric mean plasma MGX concentrations (17.9 μg/mL) for this regimen appeared to reach steady state on or before day 4 (Fig. 5, Table 4), as did the AUC_0–24_ (220 μg · h/mL). The geometric mean *C*_max_ and AUC_0–24_ on days 1, 4, and 7 were comparable (ranging from 15.7 to 17.9 μg/mL and 201 to 220 μg · h/mL, respectively), indicating no extremes in peak or overall concentrations. Comparison of exposure parameters after receiving a 600-mg dose infused over 1 h on day 7 for the loading regimen with those of a 600-mg dose infused over 3 h on day 14 without a loading regimen (Table 2) shows reasonable agreement when the difference in infusion times between the two dose regimens is considered (17.7 [Table 4] versus 15.4 μg/mL for *C*_max_ and 201 [Table 4] versus 245 μg · h/mL for AUC_0–24_). This suggests that the loading regimen meets the objective of reaching steady state within the first 24 h of dosing.

**TABLE 4:** MGX after i.v. infusion of a loading regimen^*a*^

Study 1 (i.v.), loading dose cohort			Pharmacokinetic parameter (geometric %CV)					
No.	FMGX dose	*n*	Study day	*C*_max_ (μg/mL)	AUC_0–24_ (μg · h/mL)	*t*_1/2_ (h)	CL (mL/h/kg)	*V*_z_ (L/kg)
12	1,000 mg/2h at 0 and 9 h	5	1	15.7 (25.2)	211 (19.9)	^*b*^	^*b*^	^*b*^
	600 mg/1 h QD	5	4	17.9 (11.6)	220 (5.58)	^*b*^	^*b*^	^*b*^
		5	7	17.7 (9.14)	201 (13.3)	48.0 (41.4)^*c*^	33.4 (14.4)	2.27 (34.4)^*c*^

a Data are presented for study 1 as the geometric mean (geometric %CV) for the PK population. %CV, percent coefficient of variation; AUC_0–24_, area under the concentration-time curve from time zero to 24 h postdose; CL, clearance; *C*_max_, maximum plasma concentration; FMGX, fosmanogepix; i.v., intravenous; MGX, manogepix; *n*, number of participants; QD, once daily; *t*_1/2_, terminal phase half-life; *V*_z_, volume of distribution.

b Parameter not applicable to the study day.

c *n* = 4.

### Pharmacokinetics of single and multiple ascending oral FMGX doses.

**FIG 6:**
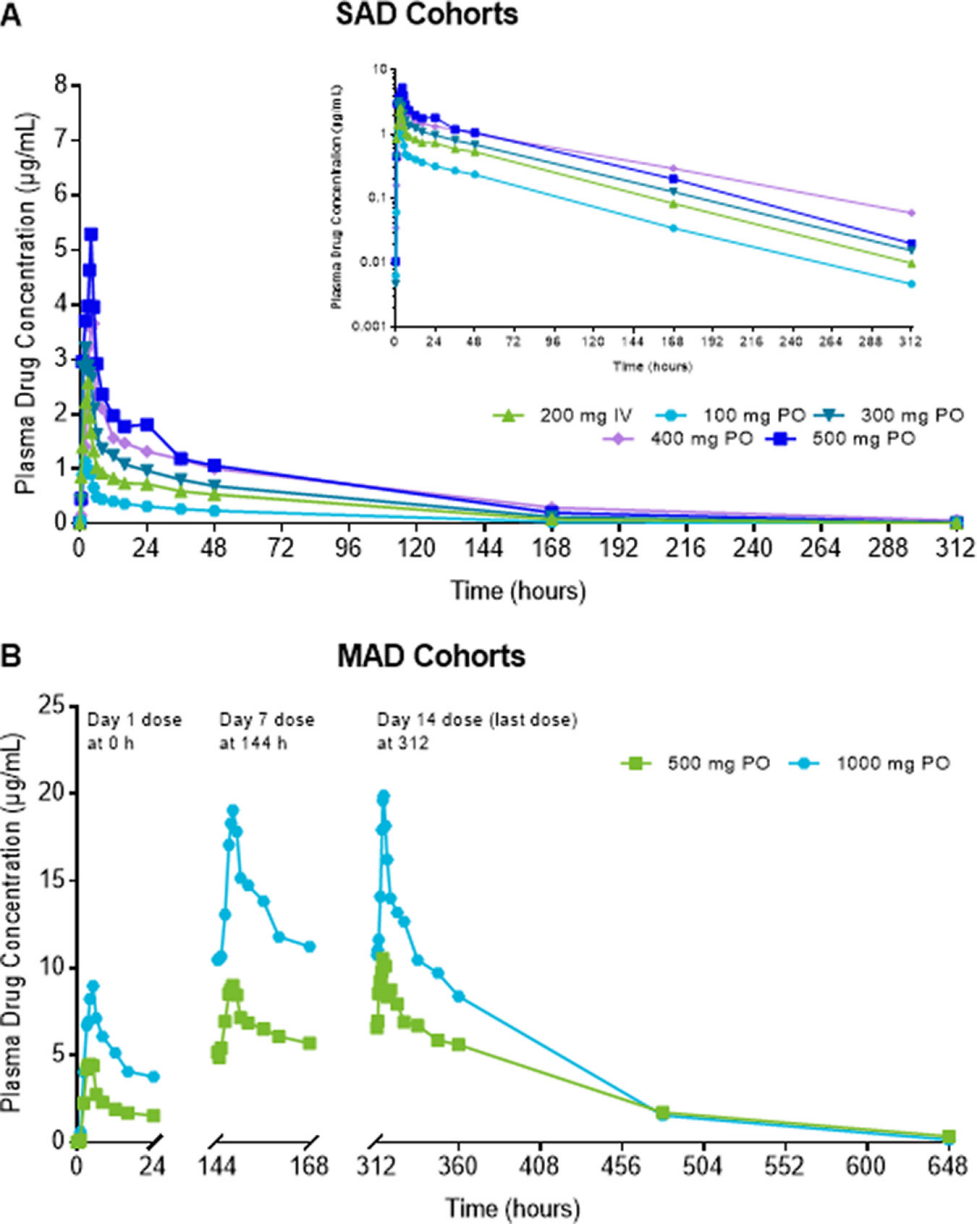
MGX concentration profile after single and multiple ascending oral FMGX doses. (A) SAD cohorts; (B) MAD cohorts. Data are presented for study 2 cohorts 1a and 1b (A) and cohorts 2 and 3 (B) as geometric mean MGX plasma concentrations for participants in the PK population (*n* = 8 for 400 mg p.o., *n* = 6 for all other treatment groups) using linear (panel A, main figure and panel B) and semilogarithmic (panel A, inset) scales. A single i.v. dose for bioequivalence was infused over 3 h; doses were administered daily for the MAD segment. The SAD and MAD phases were conducted under *ante cibum* and *post cibum* conditions, respectively. FMGX, fosmanogepix; n, number of participants; MAD, multiple ascending dose; MGX, manogepix; PO, oral, SAD, single ascending dose.

The graph of the MGX PK profile after oral administration of FMGX was comparable to that after i.v. infusion of FMGX (Fig. 6). As observed with i.v. dosing, there were dose-proportional increases in the geometric mean MGX plasma concentrations and values for *C*_max_ and AUC with single and repeat oral dosing (Fig. 6, Table 5, see Fig. S1 in the supplemental material). Although conditions for dosing changed from *ante cibum* for the SAD phase to *post cibum* for the MAD phase to improve tolerability, the food effect evaluation described below suggests this change may not significantly impact exposure.

**TABLE 5:** MGX pharmacokinetic parameters after single and multiple ascending oral FMGX doses^*a*^

Study 2 (i.v. and p.o.) SAD cohorts						
Cohort			Pharmacokinetic parameter (geometric %CV)			
No.	FMGX dose	*n*	Study day	*C*_max_ (μg/mL)	AUC_∞_ (μg · h/mL)	*t*_1/2_ (h)
1a	200 mg i.v.	6	1	2.64 (12.1)	87.5 (9.85)	49.1 (28.9)
1a	100 mg p.o.	6	1	1.30 (22.7)	39.6 (13.4)	49.5 (27.7)
1a	300 mg p.o.	6	1	3.75 (23.9)	122 (10.1)1	52.5 (35.2)
1a	400 mg p.o.	8	1	4.25 (21.9)	177 (15.8)	64.7 (29.0)
1a	500 mg p.o.	6	1	6.41 (21.0)	205 (13.7)	44.9 (32.6)
1b	400 mg p.o. *ante cibum*	8	1	4.25 (21.9)	177 (15.8)	64.7 (29.0)
1b	400 mg p.o. *post cibum*	8	1	4.52 (35.3)	188 (14.6)	67.5 (24.2)
Study 2 (p.o.) MAD cohorts (noncompartmental analysis)						
Cohort			Pharmacokinetic parameter (geometric %CV)			
No.	FMGX dose (mg p.o.)	*n*	Study day	*C*_max_ (μg/mL)	AUC_0–24_ (μg · h/mL)	*t*_1/2_ (h)
2	500	6	1	6.18 (24.4)	50.8 (16.8)	
			7	10.9 (12.1)	155 (8.93)	
			14	12.0 (14.7)	191 (9.03)	73.4 (18.5)
3	1,000	6	1	11.0 (14.2)	119 (15.0)	
			7	21.3 (15.0)	316 (17.26)	
			14	21.3 (13.4)	326 (16.8)	52.6 (25.0)

a Data are presented for study 2 as the geometric mean (geometric %CV) for the PK population. The i.v. dose was infused over 3 h; doses were administered daily for the MAD segment. The SAD and MAD phases were conducted under *ante cibum* and *post cibum* conditions, respectively. %CV, percent coefficient of variation; AUC_∞_, area under the concentration-time curve from time zero to infinity; *C*_max_, maximum plasma concentration; FMGX, fosmanogepix; IV, intravenous; MAD, multiple ascending dose; MGX, manogepix; *n*, number of participants; p.o., oral; SAD, single ascending dose; *t*_1/2_, terminal phase half-life; *V*_z_, volume of distribution.

For single-dose administration, the median *T*_max_ after oral administration ranged from 2.0 to 3.75 h, compared to 3.0 h after i.v. infusion over that period. Route of administration had little effect on half-life, which ranged from 44.9 h to 67.5 h p.o. versus 49.1 h i.v. (Table 5). The geometric mean CL (22.6 mL/h/kg) and *V*_z_ (1.60 L/kg) after i.v. administration were concordant with CL/F (23.7 to 25.1 mL/h/kg) and apparent volume of distribution (*V*_z_/F) (1.62 to 2.35 L/kg) after oral administration and was independent of dose. After repeat administration, the median *T*_max_ ranged from 3.75 to 4.5 h across doses and study days, suggesting no apparent change with continued dosing, and *t*_1/2_ values were consistent with those observed previously (Table 5). The median accumulation ratio was 1.98 for *C*_max_ and 3.19 for AUC_0–24_.

### Bioavailability of MGX.

The absolute bioavailability of MGX, estimated as the dose-corrected ratio of the oral-to-i.v. AUC_0–∞_ geometric means from cohort 1a receiving 200 mg i.v. and 100 mg p.o. (Table 5), ranged from 90.6% to 101.2%, indicating essentially complete absorption of FMGX and conversion to MGX. This essentially complete oral bioavailability suggests that FMGX is a good candidate for i.v. to p.o. sequential therapy.

### Effect of food on MGX pharmacokinetics.

Oral administration of 400 mg FMGX with a high-fat/high-calorie meal had no significant effect on the exposure of MGX (Fig. S2). The least-squares geometric mean ratios were 106% for *C*_max_, area under the concentration-time curve from time 0 to time t (AUC_0–t_), and AUC_∞_, and the associated 90% confidence intervals (CIs) were within the 80% to 125% equivalence limits.

### General safety and tolerability.

All single and multiple i.v. and p.o. doses of FMGX were well tolerated. There were no severe treatment-emergent adverse events (TEAEs), serious AEs (SAEs), or withdrawals due to treatment-related TEAEs reported. No TEAEs or laboratory safety test results met any of the predefined rules that prevented dose escalation. There were no dose-limiting toxicities observed, and the maximum tolerated dose was not determined or reached in these studies. The most frequently reported drug-related TEAEs across all treatment groups were gastrointestinal disorders (nausea and vomiting), nervous system disorders (headache and dizziness), and general disorders and administration site reactions (fatigue) (Table S1), which generally were more frequent in the highest-dose groups. Tolerability of FMGX after p.o. administration generally improved under *post cibum* conditions, as TEAEs related to gastrointestinal (nausea; oral 400 mg SAD FMGX *n* [%]: *ante cibum* = 4 [50]; *post cibum* = 0 0) and nervous system disorders (headache; *ante cibum* = 3 [38]; *post cibum* = 0 0) were reduced compared to *ante cibum* conditions. No clinically significant findings were reported for the clinical laboratory parameters, electrocardiogram (ECG), physical examination, and body weight. Regarding vital signs, one participant had a body temperature of 38.5°C on day 4 prior to dosing, which led to the AE of pyrexia (considered unrelated to the study drug) and withdrawal from the study.

## DISCUSSION

Despite the high unmet medical need for new antifungal drugs with a unique mechanism of action, no new drug classes have been commercialized for IFI since the launch of the first echinocandin in 2001 (2, 27). Fungi, like humans, are eukaryotes, and consequently, most compounds toxic to fungi are also potentially toxic to the patient. Evolving antifungal drug resistance, limited spectrum of activity, and restricted routes of administration are other unresolved issues with currently available drugs (28). These limitations, along with increasing incidence of IFIs and associated mortality, highlight the urgency to expand the antifungal armamentarium.

FMGX is a first-in-class investigational antifungal prodrug. The active moiety, MGX, has a novel mechanism of action that disrupts the integrity of the cell wall through inhibition of the conserved inositol acylase Gwt1. Due to its unique mechanism of action, target-based cross-resistance with other classes of drugs (azoles, echinocandins, polyenes) is not observed. FMGX and MGX have also demonstrated broad-spectrum *in vivo* and *in vitro* activity, respectively, against clinically important yeasts and molds, including drug-resistant strains, and MGX is widely distributed within infected tissues. Importantly, MGX is distinguished from the echinocandins by significant exposure in the central nervous system (8, 15). In a study using a rabbit model of meningoencephalitis, significant reduction of the residual fungal burden in the cerebrum, cerebellum, spinal cord, meninges, and cerebrospinal fluid (CSF) at all dosages of FMGX was reported (20). This suggests that it may be a potential treatment option for fungal infections that have a propensity for dissemination into the brain tissue and CSF (8, 15).

The results presented here from two phase 1 studies that evaluated single and multiple doses of FMGX up to 1,000 mg administered via the i.v. or p.o. route highlight additional characteristics of FMGX, such as predictable linear and dose-proportional PK and maintenance of plasma exposures during the transition from i.v. to oral dosing. Oral FMGX may be administered with or without food for the convenience of ambulatory patients or those who have been discharged and continue treatment at home. Single oral dosing of FMGX demonstrated improved tolerability with food as indicated by a lower number of TEAEs related to gastrointestinal disorders (mainly nausea). At the i.v. dose levels of 10 mg to 1,000 mg and oral dose levels of 100 mg to 500 mg evaluated in these studies, FMGX use was generally well tolerated and safe, not accompanied by any serious/unexpected concerns. Most AEs were mild, transient, and resolved without intervention. No dose-limiting toxicities were observed, and no AEs or laboratory safety test results met any of the predefined criteria that prevented dose escalation. In earlier preclinical toxicity studies, no target organ toxicities were observed. Although target MGX exposures for *in vivo* efficacy against C. albicans, C. glabrata, and C. auris in a neutropenic disseminated candidiasis mouse model were generally exceeded (29), the maximum tolerated dose was not determined or reached in either study. Thus, an opportunity to explore higher exposures that may be necessary to treat more recalcitrant fungal strains exists. To address this, higher doses were subsequently evaluated in a separate clinical study, and the results will be published in a future paper.

Treatment with FMGX resulted in predictable and consistent PK profiles. Dose proportionality (linear PK) was demonstrated via high correlation between FMGX dose and MGX *C*_max_ and AUC_0–∞_ for i.v. doses with similar infusion rates and for p.o. doses (Fig. S1). Shortening the infusion period of a 1,000-mg dose from 3 h to 0.5 h resulted in increased *C*_max_ but had a minimal effect on tolerability (Table 3, Fig. 4). Total drug exposures that were associated with antifungal efficacy in mouse animal models (29, 30) were achieved successfully (safe and well tolerated) in this phase 1 study.

FMGX has high oral bioavailability (>90%), making it an ideal candidate for developing i.v. and p.o. formulations. The i.v. therapy may be more appropriate for a patient who is critically ill or unable to reliably absorb oral medications. However, for other patients, the oral formulation may be preferred (while switching from i.v. to oral therapy or to initiate treatment with oral medication only). For some drugs (antimicrobials such as clindamycin, cephalexin, amoxicillin, cloxacillin, and antifungal voriconazole), switching from the i.v. to p.o. route of administration has the potential to lower the plasma exposure to ineffectual levels and result in treatment failure (31, 32). However, the i.v. to oral switch of FMGX resulted in comparable exposures between the two dosing formulations; specifically, the AUC and *C*_max_ were unchanged. FMGX can be dosed QD given its long half-life of 2.5 days, and oral doses do not require administration with food, resulting in a simple dosing regimen that can facilitate patient adherence to treatment.

Taken together, these drug characteristics suggest that FMGX could be a novel antifungal treatment option for patients with life-threatening IFIs. Other open-label, multicenter studies to evaluate FMGX in patients undergoing chemotherapy for acute myeloid leukemia with neutropenia (NCT03333005), nonneutropenic patients with candidemia (NCT03604705), and patients with candidemia/invasive candidiasis caused by C. auris (NCT04148287) are completed, and results will be published separately.

## MATERIALS AND METHODS

### Study design.

**FIG 7:**
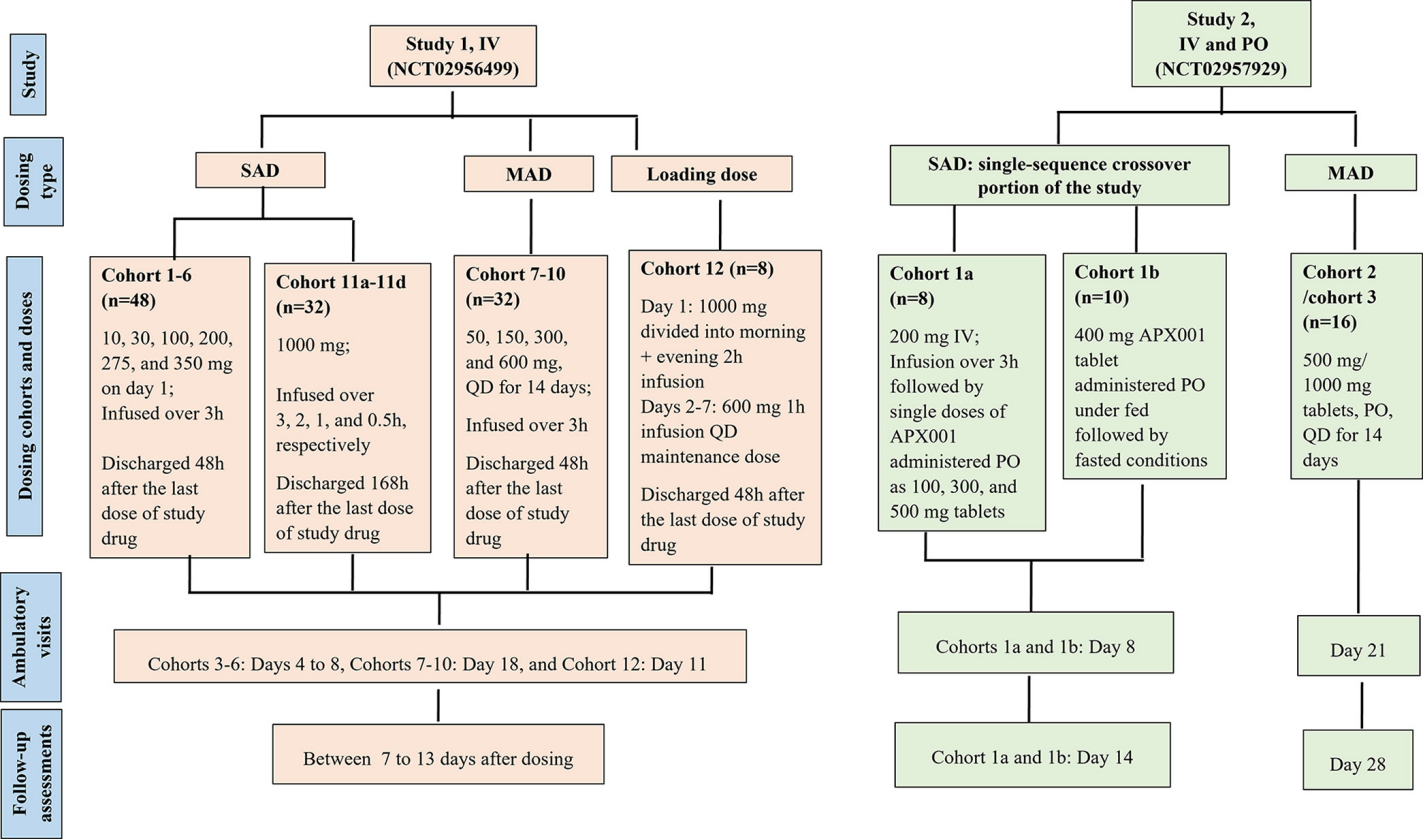
Study design. IV, intravenous; MAD, multiple ascending dose; n, number of patients; PO, oral; QD, once daily; SAD, single ascending dose.

The studies of FMGX (APX001-101 [study 1; NCT02956499] and APX001-102 [study 2; NCT02957929]) (Fig. 7) described below were conducted in accordance with the principles of the Declaration of Helsinki and followed International Conference on Harmonisation (ICH) Good Clinical Practice (GCP) guidelines (33, 34). The protocols were reviewed and approved by an Independent Ethics Committee (IEC) of the foundation “Evaluation of Ethics in Biomedical Research,” The Netherlands, before initiating each clinical study. All participants provided written informed consent prior to participating.

A safety review committee reviewed safety and PK data from all participants to determine the appropriateness of dose escalation for both studies as well as the safety and PK data from the two sentinel participants in each cohort to determine the appropriateness of continued dosing of FMGX.

**(i) Study 1.** This was a first-in-human, phase 1, randomized, double-blind, placebo-controlled, single-ascending dose (SAD) and multiple-ascending dose (MAD) study sponsored by Amplyx Pharmaceuticals and conducted by ICON (formerly PRA Health Sciences), Groningen, The Netherlands, from May 2016 to July 2017 to investigate the safety, tolerability, and PK of FMGX administered i.v. Eight participants in each cohort were randomized to receive either FMGX or placebo in a 6:2 ratio. This study included three sets of cohorts, SAD cohorts (1 to 6), MAD cohorts (7 to 10), and different dosing regimen cohorts (11 and 12). Cohort 11 was further divided into cohorts 11a to 11d, and cohort 12 was a loading dose cohort. SAD cohorts 1 to 6 received single doses of 10, 30, 100, 200, 275, and 350 mg, respectively, once on day 1. MAD cohorts 7 to 10 received doses of 50, 150, 300, and 600 mg, respectively, once daily (QD) for 14 days. Doses were infused over 3 h for cohorts 1 to 10, based on animal toxicology studies. Cohorts 11a to 11d and cohort 12 evaluated different dosing regimens, including various infusion times and a loading dose strategy. Cohorts 11a to 11d received single doses of 1,000 mg infused over 3, 2, 1, and 0.5 h, respectively. Cohort 12 received a loading dose of 1,000 mg divided into a morning 2-h infusion and an evening 2-h infusion on day 1 (with 9 h between the start of the sequential infusions) and a 600-mg 1-h infusion QD maintenance dose on days 2 to 7.

All participants were admitted to the clinic the day prior to the first dose of the study drug. Participants from all cohorts except for cohort 11 were discharged 48 h after the last dose of study drug administration, after completion of study assessments. Cohort 11 participants were discharged 168 h after the start of single study drug infusion. Ambulatory visits were required for Cohorts 3 to 6 (days 4 to 8), cohorts 7 to 10 (day 18), and cohort 12 (day 11). Timing of the follow-up assessments varied between cohorts, but they were performed between 7 and 13 days after dosing.

The study drug was administered between 0800 and 1100 h after a fast of ≥10 h (standardized for i.v. and oral doses) and, as applicable, approximately the same time (±30 min) on each dosing day. For cohort 12, an additional dose on day 1 was administered between 1700 and 1800 h.

**(ii) Study 2.** This was a phase 1, randomized, double-blind, placebo-controlled, SAD and MAD study sponsored by Amplyx Pharmaceutical and conducted by ICON (formerly PRA Health Sciences) Groningen, The Netherlands, from November 2016 to March 2017 to investigate the safety, tolerability, PK, bioavailability, and food effect of single doses of FMGX administered i.v. (for purposes of establishing absolute bioavailability) and p.o.

This study included two parts, single-sequence crossover (SAD: comprising cohorts 1a and 1b) and MAD (cohorts 2 and 3). The final cohort 4 is not included here and will be reported separately, since it focused on a different aspect (assessment of the drug-drug interactions [DDI] potential of FMGX). Cohorts 1a and 1b were the single-sequence crossover part of the study. Eight participants in cohort 1a were randomized to receive either FMGX or placebo in a 6:2 ratio in the following sequence: 200-mg i.v. infusion over 3 h followed by single p.o. FMGX doses of 100, 300, and 500 mg, administered in tablet form. Participants in cohort 1b (*n* = 10) were randomized to receive either FMGX or placebo in an 8:2 ratio as a single p.o. FMGX dose of 400-mg administered in tablet form under *post cibum* (fed) conditions followed by *ante cibum* (fasted) conditions. For *post cibum* conditions, the dose was administered 30 min after the start of a standardized breakfast. For *ante cibum* conditions, the dose was administered after an overnight fast of at least 10 h, which continued for a period of ~4 h after drug administration.

For each period, participants in cohorts 1a and 1b were domiciled from days −1 to 3, had an ambulatory visit on day 8, and had follow-up assessments performed on day 14 ± 2 (last period only). Each period in cohorts 1a and 1b was separated by 14 days.

Cohorts 2 and 3 were the multiple-dose portion of the study. Eight participants in each cohort were randomized to receive either FMGX or placebo in a 6:2 ratio as single p.o. FMGX doses of 500- or 1,000-mg, (for cohorts 2 and 3, respectively) administered in tablet form QD for 14 days under *post cibum* conditions. Participants in cohorts 2 and 3 were domiciled from days −1 to 16, had an ambulatory visit on day 21, and had follow-up assessments performed on day 28 ± 2.

The study drug for all cohorts was administered between 0800 and 1100 h and, as applicable, around the same time on each dosing day (±30 min).

### Eligibility criteria.

Eligible participants were male or female, 18 to 55 years of age, with a body mass index (BMI) of 18 to 30 kg/m^2^. Female (either surgically sterile or agreed to use birth control) and male (with partner of childbearing potential who agreed to use appropriate barrier contraception) participants were medically fit with no clinically significant medical history, physical examination results, laboratory profiles, vital signs, or electrocardiograms (ECGs), as deemed by the investigator. Tobacco use within 6 months and medications within 7 to 14 days of first dose were prohibited to avoid any possible adverse effects from acute and chronic smoking-related diseases confounding the assessment of investigational medicinal product safety signal detection. Participation in more than one cohort or prior exposure to FMGX was also exclusionary.

### Pharmacokinetic assessment.

**(i) Study 1.** PK collection times for SAD cohorts 1 to 6 were predose and 0.5, 2, 3, 3.5, 4, 5, 6, 8, 12, 16, 24, 36, 48, 72, 96, 120, 144, 168, and 240 h (±48 h) postdose (defined as the starting point of infusion for all cohorts receiving i.v. infusion). Blood collection times for cohorts 1 and 2 were shorter and ended 48 h postdose with an additional collection 168 h postdose for cohort 2. PK collection times for cohorts 7 to 10 were the same as for cohorts 3 to 6 through 24 h postdose for days 1 and 14; predose and 2, 3, 4, 8, and 24 h postdose for day 7; and 36 and 48 h postdose on day 14. Cohorts 11a to 11d each had a predose sample collected, but PK sampling for the first 2.5 h postdose was more frequent for cohorts with shorter infusion times: 0.5 and 2 h postdose for cohort 11a; 0.5, 1, 1.5, 2, and 2.5 h postdose for cohort 11b; 0.33, 0.67, 1, 1.5, and 2 h postdose for cohort 11c; and 0.17, 0.33, 0.5, 0.75, 1, and 2 h postdose for cohort 11d. PK collection times for cohorts 11a to 11d from 3 to 240 ± 48 h postdose mirrored those from cohorts 3 to 6. PK collection times for cohort 12 were predose and 0.5, 1, 3, 6, 9, 10, 11, 16, 18, and 24 h postdose for day 1; predose and 0.5, 1, 3, 6, 8, and 24 h postdose for day 4; and predose and 0.5, 1, 2, 3, 4, 6, 8, 12, 16, 24, 36, and 48 h postdose on day 7.

**(ii) Study 2.** PK collection times for cohorts 1a and 1b were predose and 0.5, 1, 2, 3, 3.5, 4, 5, 6, 8, 12, 16, 24, 36, 48, 168, and 336 h postdose. For cohorts 2 and 3, PK collection times were the same as for cohorts 1a and 1b through 24 h postdose on days 1, 7, and 14 and were 36, 48, 168, and 336 h postdose on day 14.

### Safety assessment.

Safety and tolerability assessments included monitoring adverse events (AEs), vital signs, 12-lead electrocardiograms (ECG), physical examinations, body weight, and clinical laboratory evaluations. Clinical laboratory samples were collected after a fasting period of ≥4 h and analyzed for clinical chemistry, hematology, urinalysis, and coagulation.

The severity of all the AEs was graded by using the NCI CTCAE version 4.0. The intensity of the AE was graded as mild, moderate, or severe. Any severe or very severe AEs that occurred during the study were categorized under the CTCAE severe category. Dose escalation was to be stopped based on predefined dose escalation stopping rules, which were either two participants with study drug-related grade 3/4 AE or one participant with study drug-related SAE, a possibility of an inappropriate safety risk, or two participants experiencing study drug-related grade 2 AE classified as nervous system disorder.

### Bioanalysis.

The analysis of FMGX and MGX in acidified EDTA 4-mL plasma samples was performed at the Bioanalytical Laboratory of ICON (formerly PRA Health Sciences, Assen, The Netherlands) using a validated liquid chromatography-mass spectrometry/mass spectrometry (LC-MS/MS) method. Quality control procedures and acceptance criteria were based on the U.S. FDA Guidance for Industry-Bioanalytical Method Validation (35), a conference report (36), and the EMA guideline on bioanalytical method validation (37). Additional stability validation experiments for FMGX were performed using bench-top stability, freeze/thaw stability, and long-term frozen stability. The results show that FMGX at 40,000 ng/mL in plasma was stable up to 25 h at room temperature, up to 5 cycles in plasma at −20°C, and up to 118 days in plasma at −20°C, respectively. The lower limit of quantitation for both analytes was 0.5 ng/mL.

### Statistical analysis.

Participant demographics, characteristics, safety, and PK data were summarized descriptively by study part and treatment. Plasma PK parameters were estimated using noncompartmental analysis. Dose proportionality for *C*_max_ and AUC_0–∞_ was compared across each dose level using a nonlinear regression model. All statistical analyses were performed using SAS version 9.4 or higher. The safety set included all participants who received ≥1 dose of study drug. The PK set included all participants who received ≥1 dose of FMGX and provided sufficient bioanalytical assessments to calculate reliable estimates of the PK parameters.
