# Supplementary material for: Safety and Pharmacokinetics of Intravenous and Oral Fosmanogepix, a First-in-Class Antifungal Agent, in Healthy Volunteers
Source: Antimicrob Agents Chemother. 2023 Mar 29;67(4):e01623-22. doi: 10.1128/aac.01623-22 (PMC10112065; doi:10.1128/aac.01623-22)
Supplement: Supplemental file 1 — Supplemental material. Download aac.01623-22-s0001.pdf, PDF file, 0.2 MB [file aac.01623-22-s0001.pdf]

Supplemental material for publication

Supplemental figure legends

Supplemental figure 1: Relationship between the (A) C_{max} and (B) $AUC_{(inf)}$ and dose of MGX after single oral dose of FMGX in participants under *ante cibum* conditions (SAD cohort)

Abbreviations: AUC_{inf} = area under the concentration-time curve from time zero to infinity; C_{max} = maximum plasma concentration; FMGX = fosmanogepix; n = number of participants; MGX = manogepix; SAD = single ascending dose.

Data are presented for Study 2 Cohort 1a as geometric mean MGX plasma concentrations for participants in the PK population (n = 6-8 per group) using a linear scale.

Supplemental figure 2. Single-dose MGX concentration profile under *post cibum* and *ante cibum* conditions

Abbreviations: MGX = manogepix; n = number of participants; PK = pharmacokinetics; PO = oral.

Data are presented for Study 2 Cohort 1b as geometric mean MGX plasma concentrations for participants in the PK population (n = 8 per group) using a linear scale.

For the *post cibum* condition, the dose was administered 30 minutes after the start of a standardized breakfast.

Supplemental figure 1: Relationship between the (A) C_{max} and (B) $AUC_{(inf)}$ and dose of MGX after single oral dose of FMGX in participants under *ante cibum* conditions (SAD cohort)

A. C_{max} **B. $AUC_{(inf)}$**

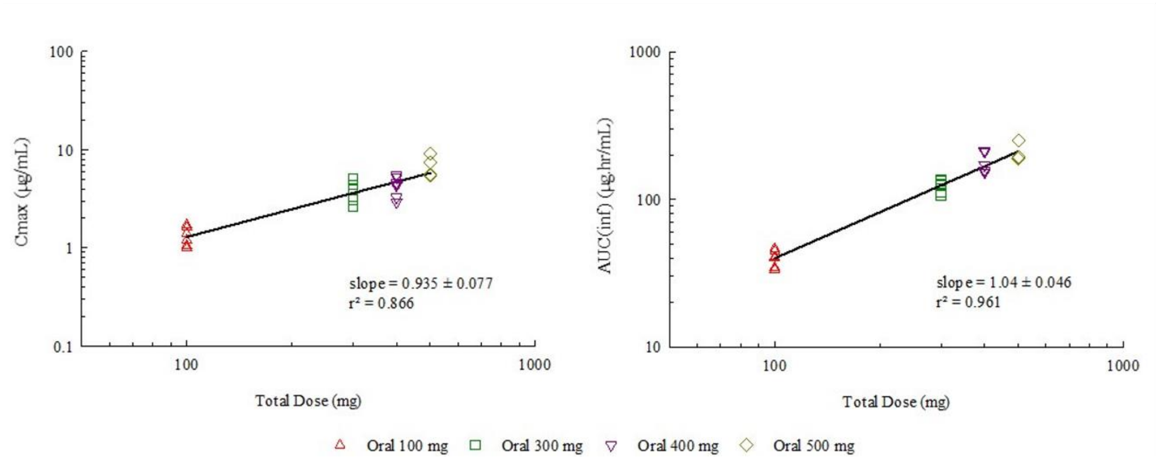

Supplemental figure 2. Single-dose MGX concentration profile under *post cibum* and *ante cibum* conditions

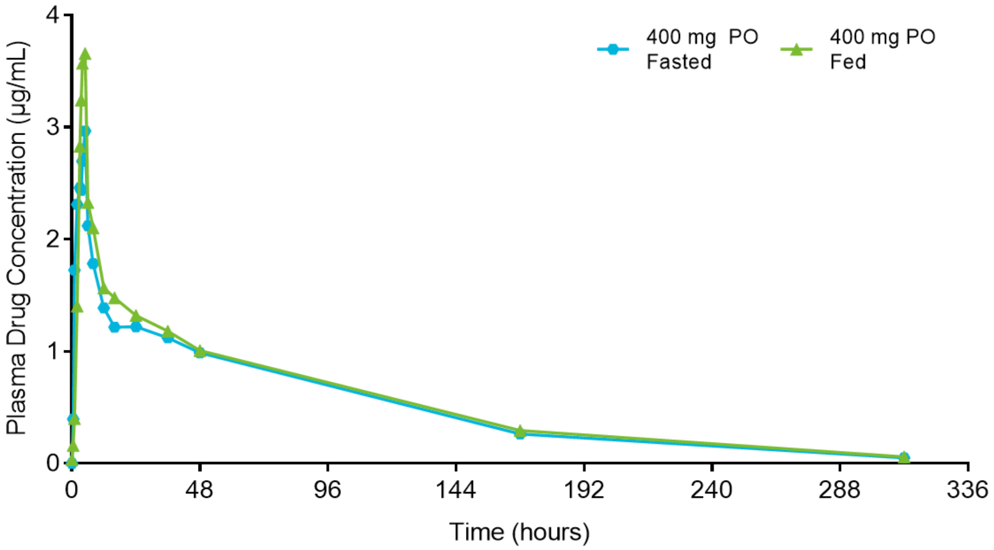

Supplemental table 1. Drug-related treatment-emergent adverse events by system organ class and preferred terms during Study 1 and Study 2

	**Study 1** (IV)								**Study 2** (IV and PO)						
	Cohorts 1-6		Cohort 7-10		Cohorts 11a-11d		Cohort 12		Cohort 1a		Cohort 1b			Cohorts 2 and 3	
Preferred Term E/n (%)	PBO n=12	10-350 mg n=36	PBO n=8	50-600 mg n=24	PBO n=8	1000 mg n=24	PBO n=2	1000→ 600 mg n=6	PBO n=2	100-500 mg n=6	PBO n=2	400 mg *Ante cibum* n=8	400 mg *Post cibum* n=8	PBO n=4	500 & 1000 mg n=12
Any	2/2 (17)	4/3 (8)	0	27/5 (21)	0	29/12 (50)	1/1 (50)	19/6 (100)	0	0	1/1 (50)	8/5 (63)	0	0	79/6 (50)
Gastrointestinal disorders	1/1 (8)	2/1 (3)	0	11/2 (8)	0	9/6 (25)	0	12/6 (100)	0	0	0	5/4 (50)	0	0	61/6 (50)
Diarrhoea	0	0	0	0	0	0	0	0	0	0	0	1/1 (13)	0	0	0
Nausea	1/1 (8)	2/1 (3)	0	7/1 (4)	0	6/6 (25)	0	9/6 (100)	0	0	0	4/4 (50)	0	0	42/6 (50)
Vomiting	0	0	0	1/1 (4)	0	3/3 (13)	0	3/3 (50)	0	0	0	0	0	0	18/5 (42)
Dyspepsia	0	0	0	0	0	0	0	0	0	0	0	0	0	0	1/1 (8)
Abdominal discomfort	0	0	0	3/1 (4)	0	0	0	0	0	0	0	0	0	0	0
Nervous system disorders	1/1 (8)	2/2 (6)	0	6/3 (13)	0	18/10 (42)	0	4/3 (50)	0	0	1/1 (50)	3/3 (38)	0	0	1/1(8)
Headache	1/1 (8)	1/1 (3)	0	1/1 (4)	0	7/4 (17)	0	2/2 (33)	0	0	1/1 (50)	3/3 (38)	0	0	0

Somnolence	0	1/1 (3)	0	0	0	0	0	0	0	0	0	0	0	0	0
Dizziness	0	0	0	2/1 (4)	0	8/7 (29)	0	2/2 (33)	0	0	0	0	0	0	0
Head discomfort	0	0	0	3/2 (8)	0	0	0	0	0	0	0	0	0	0	0
Dysgeusia	0	0	0	0	0	2/2 (8)	0	0	0	0	0	0	0	0	0
Presyncope	0	0	0	0	0	1/1 (4)	0	0	0	0	0	0	0	0	0
Metabolism and nutrition disorders	0	0	0	0	0	0	0	0	0	0	0	0	0	0	2/1 (8)
Decreased appetite	0	0	0	0	0	0	0	0	0	0	0	0	0	0	2/1 (8)
General disorders and administration site reactions	0	0	0	5/3 (13)	0	2/2 (8)	1/1 (50)	3/2 (33)	0	0	0	0	0	0	14/5 (42)
Fatigue	0	0	0	0	0	0	0	0	0	0	0	0	0	0	13/5 (42)
Feeling hot	0	0	0	1/1 (4)	0	0	0	0	0	0	0	0	0	0	1/1 (8)
Chest discomfort	0	0	0	4/3 (13)	0	0	0	0	0	0	0	0	0	0	0
Infusion site haematoma	0	0	0	0	0	1/1 (4)	0	0	0	0	0	0	0	0	0

Infusion site pain	0	0	0	0	0	1/1 (4)	0	1/1 (17)	0	0	0	0	0	0	0
Infusion site irritation	0	0	0	0	0	0	1/1 (50)	2/2 (33)	0	0	0	0	0	0	0
Vascular disorder	0	0	0	3/2 (8)	0	0	0	0	0	0	0	0	0	0	0
Hot flush	0	0	0	3/2 (8)	0	0	0	0	0	0	0	0	0	0	0
Respiratory, thoracic and mediastinal disorders	0	0	0	1/1 (4)	0	0	0	0	0	0	0	0	0	0	0
Painful respiration	0	0	0	1/1 (4)	0	0	0	0	0	0	0	0	0	0	0
Injury, poisoning and procedural complications	0	0	0	1/1 (4)	0	0	0	0	0	0	0	0	0	0	1/1 (8)
Procedural vomiting	0	0	0	0	0	0	0	0	0	0	0	0	0	0	1/1 (8)
Procedural nausea	0	0	0	1/1 (4)	0	0	0	0	0	0	0	0	0	0	0

Abbreviations: E = events; FMGX = fosmanogepix; IV = intravenous; n = number of participants; PBO = placebo; PO = oral.
Data are events/n (%) of participants in the Safety Population. All events reported in the FMGX treatment group in Cohorts 7-10 and Cohorts 2 and 3 were in the 600 mg and 1000 mg groups, respectively.
